# Treatment plan prescreening for patient-specific quality assurance measurements using independent Monte Carlo dose calculations

**DOI:** 10.3389/fonc.2022.1051110

**Published:** 2022-11-07

**Authors:** Yuan Xu, Ke Zhang, Zhiqiang Liu, Bin Liang, Xiangyu Ma, Wenting Ren, Kuo Men, Jianrong Dai

**Affiliations:** Department of Radiation Oncology, National Cancer Center/National Clinical Research Center for Cancer/Cancer Hospital, Chinese Academy of Medical Sciences and Peking Union Medical College, Beijing, China

**Keywords:** patient-specific QA, dosimetric verification, Monte Carlo, independent dose verification, ArcherQA

## Abstract

**Purpose:**

This study proposes a method to identify plans that failed patient-specific quality assurance (QA) and attempts to establish a criterion to prescreen treatment plans for patient-specific QA measurements with independent Monte Carlo dose calculations.

**Materials and methods:**

Patient-specific QA results measured with an ArcCHECK diode array of 207 patients (head and neck: 25; thorax: 61; abdomen: 121) were retrospectively analyzed. All patients were treated with the volumetric modulated arc therapy (VMAT) technique and plans were optimized with a Pinnacle v16.2 treatment planning system using an analytical algorithm-based dose engine. Afterwards, phantom verification plans were designed and recalculated by an independent GPU-accelerated Monte Carlo (MC) dose engine, ArcherQA. Moreover, sensitivity and specificity analyzes of gamma passing rates between measurements and MC calculations were carried out to show the ability of MC to monitor failing plans (ArcCHECK 3%/3 mm,<90%), and attempt to determine the appropriate threshold and gamma passing rate criterion utilized by ArcherQA to prescreen treatment plans for ArcCHECK measurements. The receiver operator characteristic (ROC) curve was also utilized to characterize the performance of different gamma passing rate criterion used by ArcherQA.

**Results:**

The thresholds for 100% sensitivity to detect plans that failed patient-specific QA by independent calculation were 97.0%, 95.4%, and 91.0% for criterion 3%/3 mm, 3%/2 mm, and 2%/2 mm, respectively, which corresponded to specificities of 0.720, 0.528, and 0.585, respectively. It was shown that the 3%/3 mm criterion with 97% threshold for ArcherQA demonstrated perfect sensitivity and the highest specificity compared with other criteria, which may be suitable for prescreening treatment plans treated with the investigated machine to implement measurement-based patient-specific QA of patient plans. In addition, the area under the curve (AUC) calculated from ROC analysis for criterion 3%/3 mm, 3%/2 mm, and 2%/2 mm used by ArcherQA were 0.948, 0.924, and 0.929, respectively.

**Conclusions:**

Independent dose calculation with the MC-based program ArcherQA has potential as a prescreen treatment for measurement-based patient-specific QA. AUC values (>0.9) showed excellent classification accuracy for monitoring failing plans with independent MC calculations.

## 1 Introduction

The accuracy of radiotherapy is crucial to its therapeutic effects on patients. Patient-specific QA is an important standard process to identify discrepancies between the dose calculated by a treatment planning system (TPS) and that delivered by the treatment machine (1–3). For two-dimensional (2D) or three-dimensional (3D) conformal radiation therapy, the shaped fields are relatively simple to deliver and are commissioned closely to an accurate TPS model. With the introduction of intensity-modulated radiotherapy (IMRT) and volumetric modulated arc radiotherapy (VMAT), the complexity of plans has increased markedly with the different degree of modulation (4, 5). Hence, robust dosimetric verification of small fields is required to validate whether the small-field modeling is sufficiently accurate and close to the delivered dose (6). Moreover, dynamic treatments (movable multi-leaf collimator, gantry or variable intensity during beam delivering) also need to accurately verify whether or not IMRT or VMAT plans are physically achievable (3). Therefore, pretreatment patient-specific QA of treatment plans has been recommended by many reports and professional organizations to ensure safety and to find any possible clinically related errors (7–9).

Patient-specific QA can be divided into two categories: measurement-based QA and software calculation. There are many methods for measurement-based patient-specific QA, such as point dose measurements, planar dose measurements, or three-dimensional measurements with numerous devices, like ion chambers (10), films (11), diode arrays (12), or electronic portal imaging devices (EPIDs) (13), etc. However, these measurement processes are known to be time-consuming and tedious, while occupying precious machine time. Software calculation is another common approach for patient-specific QA by exporting DICOM files (14, 15) from TPS or log files (16), etc., from a treatment machine to independent programs to recalculate dose or monitor unit (MU) with different algorithms. Apparently, automated software calculation is a more effective way for patient-specific QA compared to measurements. It was reported that independent computer calculations might take the place of measurements by analyzing point dose data using a statistical process control (17). (Sochi et al.) analyzed 100 IMRT plans and concluded that an in-house independent dose calculation algorithm performed better than film measurements in predicting the plan disagreement (18). (Cry et al.) showed that the independent calculation was more sensitive in detecting failing plans than measurement-based QA (19). The good performance of independent calculation may be because the independent calculation is able to decouple the TPS errors and machine QA (18). It was reported that few TPS errors could be discovered by measurement-based patient-specific QA (20, 21) and most problems came from the delivering system, which should be settled by machine QA (22). Hence, it is debated whether or not measurement-based patient-specific QA should be replaced with independent calculation (23, 24). In a recent AAPM report, TG 219 published in October 2021, it was recommended to implement a secondary dose calculation or an MU check for every IMRT/VMAT plan (25). Nevertheless, measurement-based patient-specific QA is still widely utilized as a standard in most radiation therapy institutions and there are limitations for software to find problems in beam delivery before patient treatment.

This study proposes a method for monitoring plans that fail patient-specific QA with independent dose calculation and attempts to establish a criterion to prescreen treatment plans for patient-specific QA measurements. This proposed method combines the advantages of independent calculation for high accuracy, high efficiency, and no treatment machine time occupation with the merits of measurements to validate beam delivery before patient treatment and avoid serious errors previously reported by the New York Times (26). To exclude the impact of management of heterogeneities by different algorithms (27), independent dose calculations were carried out using phantom verification plans to increase detection sensitivity for failing plans measured with a phantom. An ArcCHECK (Sun Nuclear Corporation, Melbourne, USA) diode array was utilized for measurement-based dose verification. Numerous in-house or commercial programs have been developed for independent dose calculation with either analytical algorithm or MC code (25). It is well known that the MC method is regarded as the gold standard in dose calculation. Several commercial vendors provide MC-based independent dose calculation software, such as SciMoCa (Scientific RT GmbH, Munich, Germany), VERIQA (PTW Freiburg, Germany), and ArcherQA (Wisdom Technology Company Limited, Hefei, China). ArcherQA is a GPU-accelerated MC dose engine that provides 3D dose calculation and specific beam modeling (28). Accuracy and high speed of ArcherQA have been demonstrated in several publications (29, 30). In this study, ArcherQA was applied to perform independent dose calculations and attempt to detect failing plans from the ArcCHECK measurements.

## 2 Materials and methods

### 2.1 Patient and plan information

This study was approved by the institutional review ethical board and informed consent was waived. A total of 207 patients (head and neck: 25; thorax: 61; abdomen: 121) were enrolled retrospectively in this study. All patients were treated with a 6-MV photon beam delivered by an Elekta Versa HD accelerator (Elekta AB, Stockholm, Sweden) in flattering filter-free mode. The VMAT technique was used for optimization with the Pinnacle TPS (version 16.2, Philips Healthcare, Eindhoven, Netherlands). The dose calculation algorithm was set as an adaptive convolve dose engine in the TPS.

### 2.2 Prescreening treatment plan workflow for patient-specific QA measurements with independent MC calculations

**Figure 1:**
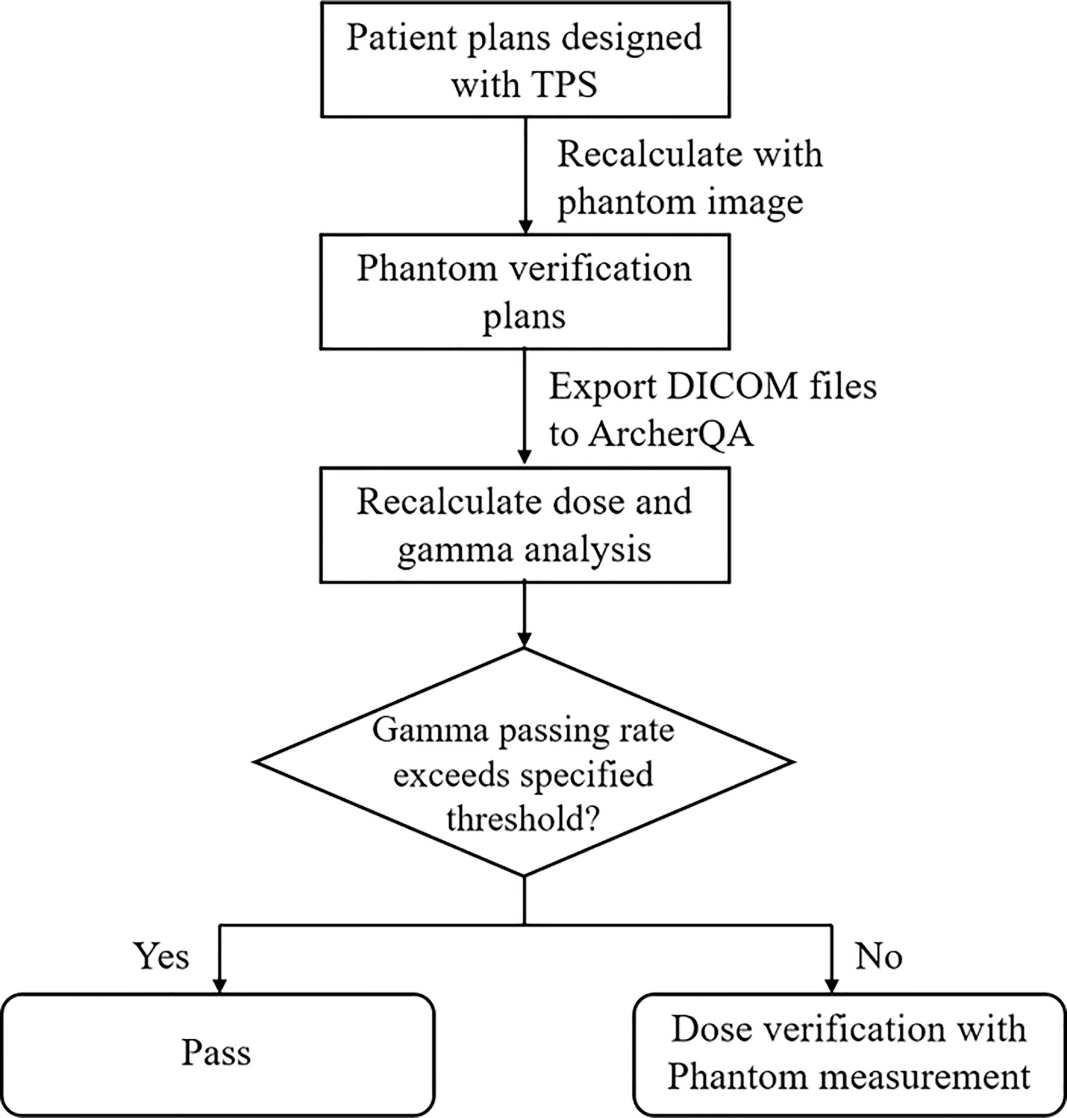
The workflow of pre-screening treatment plans for patient-specific QA measurement with ArcherQA computed with the TPS and MC. If the gamma passing rate of a plan is higher than a specific threshold, it means that the plan is acceptable (pass) for treatment; otherwise, further measurement is needed to validate the plan.

The workflow for prescreening treatment plans with MC is shown in **Figure 1**. Patient plans were initially optimized with the TPS. Phantom verification plans were then designed by recalculating dose distribution with the phantom image in the TPS and exported to ArcherQA to recalculate the dose distribution with the MC algorithm. Then, 3D gama analysis was carried out between doses computed with the TPS and MC. If the gamma passing rate of a plan is higher than a specific threshold, it means that the plan is acceptable (pass) for treatment; otherwise, further measurement is needed to validate the plan.

### 2.3 Phantom measurements

Each patient plan was recalculated on a 3D diode array ArcCHECK phantom (Sun Nuclear Corporation, Melbourne, USA) with a dose grid of 0.2 cm × 0.2 cm × 0.2 cm. The measurement data was collected with SNC patient software (version 8.2, Sun Nuclear Corporation, Melbourne, USA). The gamma passing rate was computed for evaluating dose discrepancies between the TPS and phantom measurements. The accelerator Elekta Versa HD in this study was a relatively old machine commissioned and installed in 2016 before publication of AAPM TG-218 report (31). Therefore, a dose difference of 3% and distance-to-agreement of 3 mm were chosen as the criterion with a threshold of 10% (following AAPM report TG 119) (32). A gamma passing rate lower than 90% was recognized as failing.

### 2.4 Independent MC dose calculation

#### 2.4.1 Software

The commercial MC dose engine ArcherQA (Wisdom Technology Company Limited, Hefei, China) was utilized for independent dose calculation. Electron–photon coupled transport was simulated with MC code accelerated by GPU (29). ArcherQA can perform 3D dose calculations and provide specific beam modeling and commissioning for users. The software was installed in a standard PC (Configuration: Intel Core i7–11700 @2.5 GHz, RAM 24 G, GPU NVIDIA TITAN V; Memory 12G). The calculation time was approximately one min for a head and neck dual arc VMAT plan.

#### 2.4.2 Beam modeling and phantom verification

Specific beam modeling was carried out for the Elekta Versa HD accelerator using data measured with a water tank phantom (PDD, profiles, output factors, etc.). The details for beam modeling was introduced before (28, 29). The modeling was also commissioned and validated with phantom measurement results. Forty-two phantom verification plans were recalculated with ArcherQA and the RT dose files were imported to the SNC patient software to compare with ArcCHECK measurement results. The commissioning aimed to improve the gamma passing rates between ArcherQA and ArcCHECK measurement for all these plans.

#### 2.4.3 Calculation

All 207 phantom verification plans (RT plan, RT structure, RT dose, and CT image) were imported to ArcherQA to recalculate the dose distribution with the MC algorithm. 3D gamma analysis was carried out for comparison of doses calculated by the TPS and MC algorithm with variable criteria: 3%/3 mm, 3%/2 mm and 2%/2 mm (threshold = 10%).

### 2.5 Criterion for selecting treatment plans for measurement

**Figure 2:**
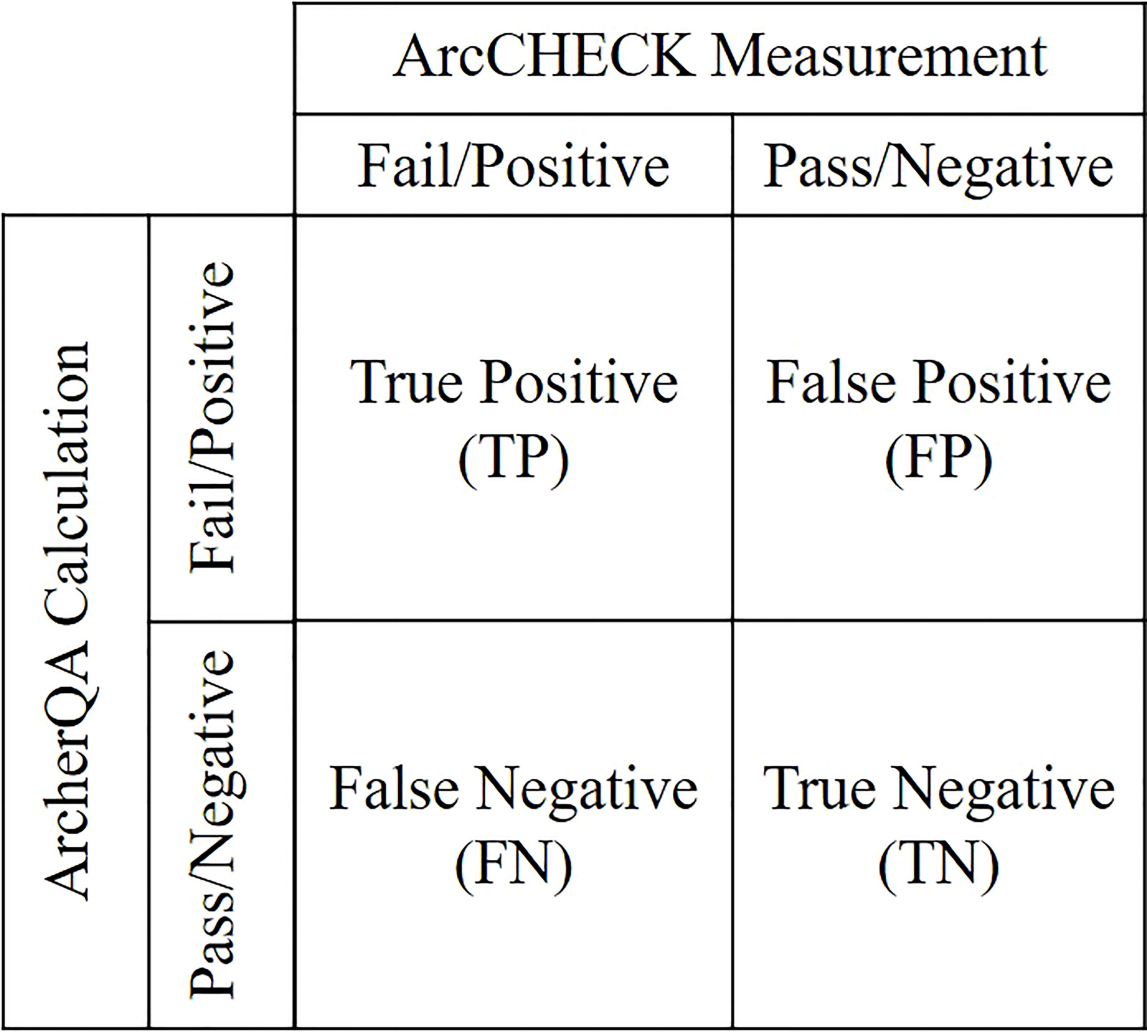
Four categories of plans charactered with ArcCHECK measurement and ArcherQA calculation.

Plans with gamma passing rates lower than the specified threshold calculated by ArcCHECK measurements or MC calculations were labeled as positive; otherwise, they were labeled as negative. For ArcCHECK measurements, the threshold was 90% under the 3%/3 mm criterion. For the MC calculation, variable thresholds were characterized with different criteria (3%/3 mm, 3%/2 mm, and 2%/2 mm). The classification accuracy was evaluated with sensitivity and specificity analyzes (19, 33–35). All plans can be divided into four categories labeled by ArcCHECK measurements and ArcherQA calculation: true positive (TP), false positive (FP), false negative (FN) and true negative (TN) as illustrated in **Figure 2**. Sensitivity was defined as the proportion of failing plans labeled by ArcCHECK measurements that were properly recognized as positive by MC calculation $Sensitivity=\frac{N(TP)}{N(TP)+N(FN)}x\;100\%$ Specificity was the proportion of negative (passing) plans labeled by ArcCHECK measurements that were properly recognized as negative by MC calculation $\text{(Specificity}=\frac{\text{N}(\text{TN})}{\text{N}(\text{TN})+\text{N}(\text{FP})})$ . The values of sensitivity or specificity can range from 0 to 1, and a sensitivity or specificity value close to one demonstrates high classification performance.

The threshold to select treatment plans for patient-specific QA measurements was defined as the minimum gamma passing rate to detect failing plans with 100% sensitivity. Moreover, appropriate criteria (3%/3 mm, 3%/2 mm, or 2%/2 mm) were selected with highest specificity when 100% sensitivity was achieved.

The ROC curve was used in evaluating the performance across different criteria (19, 33, 34). This value is plotted as the true positive rate (sensitivity) varies with false positive rate (1–specificity). The AUC was calculated to characterize the performance of the classifier. Generally, AUC values range from 0.5 to 1 (0.5 represents a random classification) and an AUC value close to one indicates a perfect classifier.

## 3 Results

### 3.1 MC beam modeling and phantom verification results

After commissioning, the average differences between measurements and ArcherQA calculations for profiles of different field size and angles were within ±2%. The average gamma passing rates between MC calculations and measurements for these 42 phantom verification plans were 3%/3 mm, 99.57% ± 0.64% and 3%/2 mm, 97.85% ± 1.71%, which was significantly higher than the average gamma passing rate between the TPS and ArcCHECK of 96.72% ± 3.44% (*p<* 0.001). These results demonstrate the high accuracy of the MC calculations.

### 3.2 Correlation analysis between ArcCHECK measurements and MC calculations

Correlation analysis between gamma passing rates calculated with measurements and those calculated with MC (variable criteria) of the 207 phantom verification plans were performed. There were 14 failing plans indicated by ArcCHECK measurements with a failing proportion of 6.76% in all plans.

**Figure 3:**
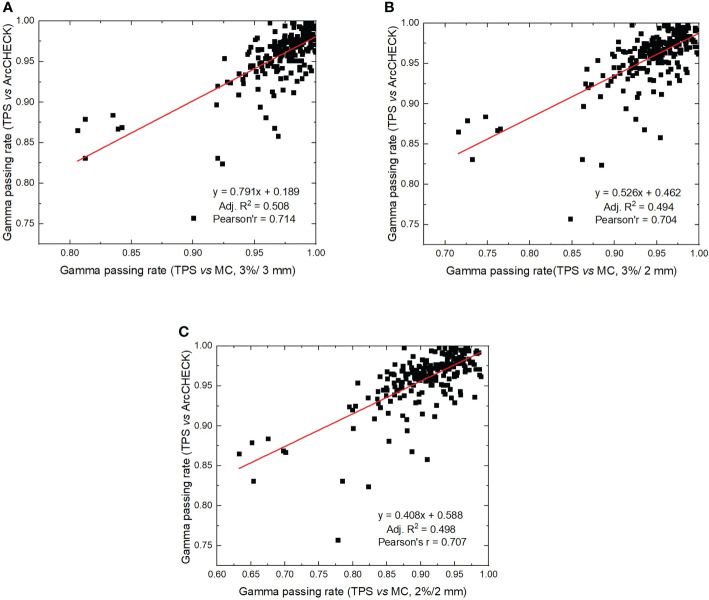
Correlation analysis between gamma passing rates calculated by ArcCHECK measurements (TPS *vs* measurements) and gamma passing rates calculated by ArcherQA (TPS *vs* MC). **(A)** 3%/3 mm criterion, **(B)** 3%/2 mm criterion, **(C)** 2%/2 mm criterion).

The average gamma passing rate for ArcCHECK measurements (3%/3 mm) was 95.82% ± 3.5%, which was significantly different from the MC calculation, with 97.14% ± 3.16% for the 3%/3 mm criterion (*p<* 0.001), 94.38% ± 4.69% for the 3%/2 mm criterion (*p<* 0.001), and 90.52% ± 6.07% for the 2%/2 mm criterion (*p<* 0.001). The correlation analysis between gamma passing rates for ArcCHECK measurements and those obtained with MC calculations with the regression method is shown in **Figure 3**. Adjusted R^2^ values were 0.508, 0.494, and 0.498 for criterion 3%/3 mm, 3%/2 mm, and 2%/2 mm with MC calculations, respectively. Pearson’s r was calculated to be 0.714, 0.704, and 0.707 for criterion 3%/3 mm, 3%/2 mm, and 2%/2 mm with MC calculations, respectively. These results shown a strong correlation (>0.7) between measurement and MC-calculated gamma passing rates (36).

### 3.3 Criterion

**Figure 4:**
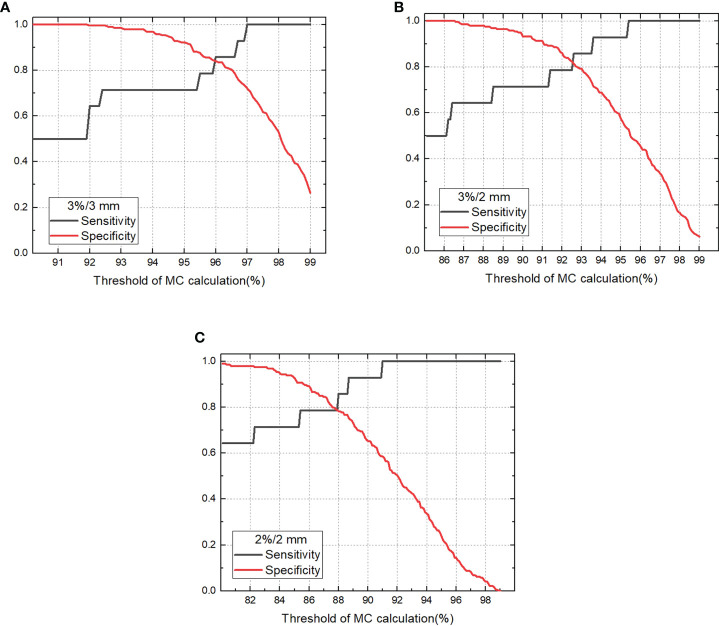
Sensitivity and specificity for detecting unacceptable plans vary with threshold for gamma passing rate of MC calculation **(A)**. 3%/3 mm criterion, **(B)** 3%/2 mm criterion, **(C)** 2%/2 mm criterion).

**Figure 5:**
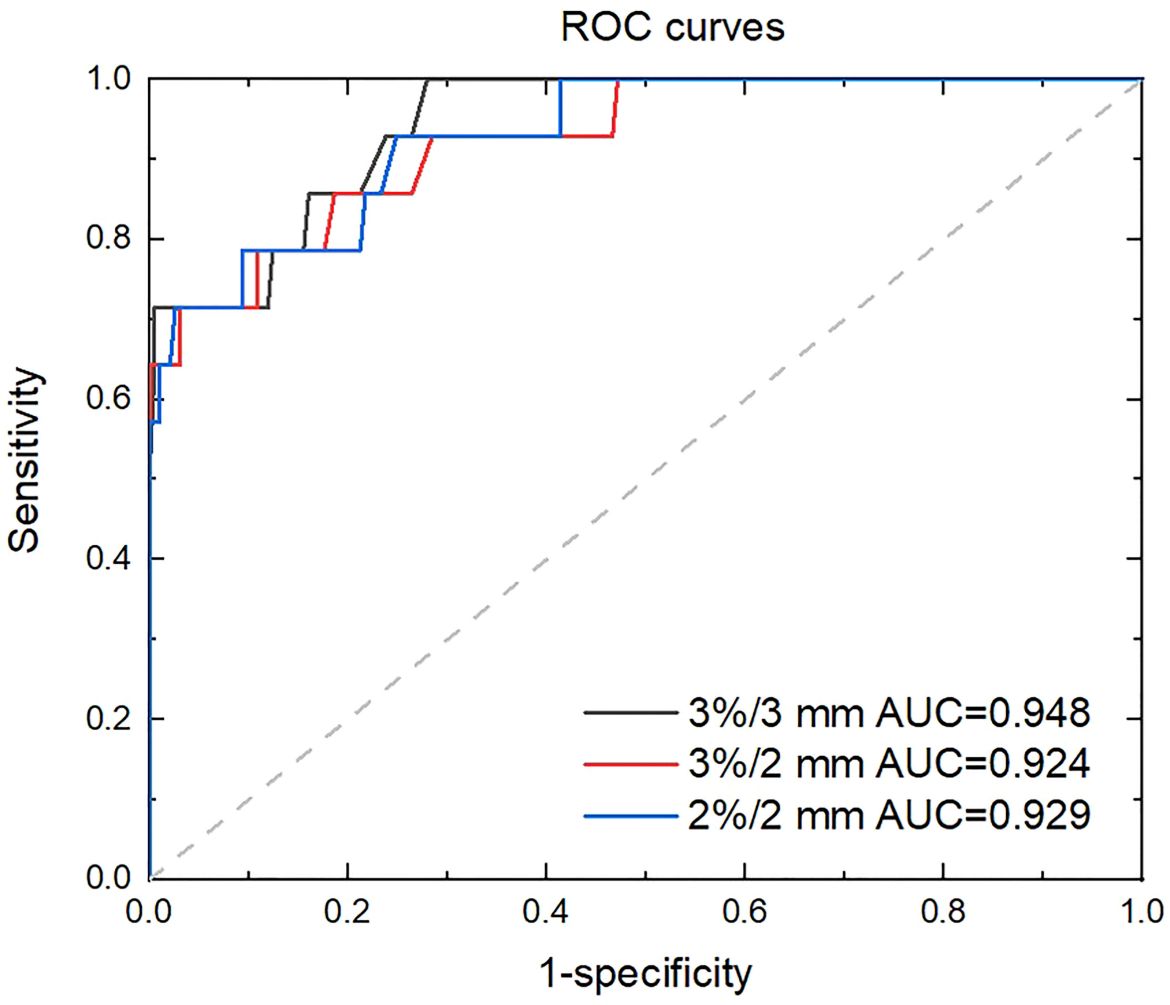
ROC curves with varying criterion of MC calculation.

The sensitivity and specificity for detecting failing plans measured with the phantom by independent MC calculation is shown in **Figure 4**. For 100% sensitivity, the thresholds were 97.0%, 95.4%, and 91.0% for criterion 3%/3 mm, 3%/2 mm, and 2%/2 mm with MC, respectively, which corresponds to specificities of 0.720, 0.528, and 0.585, respectively. For the MC calculation, the 3%/3 mm criterion showed the highest specificity compared with the other two criteria when 100% sensitivity was achieved. The ROC curves for variable criteria are plotted in **Figure 5** and the AUC indexes were 0.948, 0.924, and 0.929 for the 3%/3 mm, 3%/2 mm, and 2%/2 mm criterion calculated with MC, respectively. It is shown that all criteria indicate excellent classification accuracy with AUC >0.9. Hence, the 3%/3 mm criterion with 97% threshold may be suitable for prescreening treatment plans with the investigated machine for patient-specific measurement-based QA.

### 3.4 Validation in clinic

The criterion was tested in clinic for one month. 110 clinical treatment plans were measured with ArcCHECK as well as calculated with ArcherQA using the 3%/3 mm criterion and 97% threshold. As a consequence, one plan failed with the ArcCHECK measurement and 33 plans failed MC calculations. The plan that failed the ArcCHECK measurement was also labeled as a failing plan by the MC calculation, which showed a 100% sensitivity for detecting failing plans with MC calculations. Hence, if the MC calculation was implemented for prescreening treatment plans, approximately 385 min of machine time and labor force can be saved per month for one machine without considering phantom set up time.

## 4 Discussions

Patient-specific QA of treatment plans has been recommended for all IMRT or VMAT plans. Practically, patient-specific QA can be implemented for all plans or sampled for selected plans (37, 38). For the sampling method, the selection criteria were either totally random or based on plenty of selection criteria, such as machine availability, plan complexity, economic factors, physicist time or preference, etc. (37). So far, there is no uniform selection criterion or explicit guidelines for radiation therapy centers to screen patients for patient-specific QA (39, 40). Measurement-based and calculation-based methods are two main strategies for the patient-specific QA of patient plans. This study attempted to establish a selection criterion to prescreen treatment plans for the measurement-based patient-specific QA of patient plans with independent MC calculations, combining advantages of both a measurement-based method and software calculations. The proposed method is more efficient than delivering all patient plans for phantom verification and can still monitor delivery problems for those plans with inferior passing rates identified by MC calculation. Furthermore, it is in accordance with recommendations in AAPM TG 218 or TG 219 that an independent dose calculation could be an effective supplement to measurement-based patient-specific QA, other than replacing it (25, 31, 32).

The prerequisite to effectively monitor failing measurement plans is the accurate modeling of independent dose calculation. If the modeling of the independent calculation is more accurate than the TPS, especially for small fields or penumbral toe and tails (23), it can be more effective for independent calculation to detect TPS errors. The MC algorithm is commonly utilized in radiation dose calculation, implementing random method in the numerical simulation of interactions between particles (41, 42). ArcherQA is a commercial MC-based dose engine that mainly simulates electron or photon transport and their interactions with other primary or secondary particles (28–30). ArcherQA was evaluated with several treatment machines and validated MC codes. The differences for the percent depth doses and axial profiles were within ±3% and ±2%, respectively, compared with the benchmarked MC code EGSnrc for the Varian TrueBeam accelerator (43). With helical tomotherapy, the gamma passing rates were 99.7%, 98.5%, and 97.2% for the prostate, lung case, and head and neck cases, respectively, compared to GEANT4 (30). In this study, beam modeling was first calibrated using water tank phantom measurements and then validated with clinical plans by ArcCHECK phantom measurements. The average gamma passing rate between ArcherQA and ArcCHECK measurements (3%/3 mm) of 42 validated plans was 99.57% ± 0.64%, which was significantly higher than the average gamma passing rate between TPS and ArcCHECK of 96.72% ± 3.44% (*p<* 0.001). These results shown good modeling accuracy for MC compared to the TPS, and also a prerequisite to increase accuracy for predicting ArcCHECK results with ArcherQA

With the proposed selection method in this study, it is still crucial to determine the criterion of suitable dose differences, distance-to-agreement for gamma analysis with MC, and rational threshold for passing plans. In this study, 3%/3 mm criterion with 97% threshold was determined a suitable criterion for prescreening treatment plans with the investigated accelerator. Meanwhile, if using 97% threshold for 3%/3 mm criterion, 32.85% of patients need further measurements predicted by MC, which is a relatively reasonable proportion in selecting patients for patient-specific QA. Actually, 100% sensitivity is a relatively conservative attempt to detect all failing plans indicated by phantom measurements. As reported, 80% sensitivity might also be acceptable in clinical practice (19). The criterion was also validated in clinic with 110 treatment plans; one failed plan indicated with ArcCHECK was detected by the ArcherQA calculation, which illustrated a sensitivity of 100%. The plan failing the ArcCHECK measurement was a bilateral breast radiation VMAT plan with very high complexity and modulation. It was solved by decreasing the maximum leaf-motion speed from 1.0 cm/deg to 0.5 cm/deg, and the modified plan finally passed the ArcherQA calculation as well as the ArcCHECK measurement. This was an example for ArcherQA to monitor failed plans in clinical practice.

For the measurement, it was reported that ArcCHECK also had limitations for dependencies on beam angle, direction, field size, etc. (44, 45) It was reported that some failing plans shown by ArcCHECK could be actually acceptable (passing) as indicated by ion chamber measurements (46). This may explain why a few failing plans measured with ArcCHECK have a relatively high gamma passing rate (maximum 96.9%, 3%/3 mm criterion) calculated with MC. These plans might be actually acceptable plans that were classified as failing plans due to the measurement limitations of ArcCHECK. Nevertheless, the feasibility of ArcCHECK for patient-specific QA has been demonstrated to be acceptable in many reports (44, 45) and is widely used in clinic. The accuracy of ArcCHECK is not the main concern in this study. This study mainly aimed to monitor failing plans measured with ArcCHECK with independent dose calculation and to provide a criterion to select patients for measurements. The reasonable criterion and threshold appear to vary with treatment machine, measurement device, TPS, beam modeling, and commissioning of the MC algorithm, which need to be investigated case by case. Despite the many merits of independent dose calculation, it is still recommended to implement measurement-based patient-specific QA for hypo-fractionation, SRS, or SBRT plans for safety and strict requirements for delivering (25, 31). If the sampling method of patient-specific QA of treatment plans is utilized, independent dose calculation might be considered as an effective approach to prescreen treatment plans.

## 5 Conclusions

The feasibility of independent dose calculations with the MC-based program ArcherQA for detecting failing plans with measurement-based patient-specific QA was demonstrated. A strong correlation (>0.7) between the gamma passing rate calculated with measurements and that calculated by MC were indicated. Meanwhile, AUC values (>0.9) showed excellent classification accuracy for monitoring failed plans with independent MC calculation. Furthermore, 100% sensitivity was achieved to detect failing plans with independent MC calculation using different criteria and the 3%/3 mm criterion with 97% threshold showed the highest specificity. This criterion and threshold may be suitable for prescreening treatment plans with the investigated machine to carry out further measurement-based QA. With supplementary independent dose calculation, patient-specific QA of patient plan procedures could be more efficient and potentially more reliable.

## Data availability statement

The raw data supporting the conclusions of this article will be made available by the authors, without undue reservation.

## Ethics statement

This retrospective study was approved by the review board of Cancer Hospital, Chinese Academy of Medical Sciences, and informed consent was waived.

## Author contributions

YX and KZ: conception design, collected the data, data analysis. ZL and WR: dose verification with phantom measurements. BL and XM: dose calculation with software. JD and KM: supervised the study. All authors contributed to the article and approved the submitted version.

## Funding

This work is supported by the National Natural Science Foundation of China (Grant No.11875320) and the Beijing Nova Program (Z201100006820058).

## Acknowledgments

We thank Prof. Dr. X. George Xu, Prof. Dr. Xi Pei and Dr. Bo Cheng from University of Science and Technology of China and Anhui Wisdom Technology Company Limited for support in software and beam modelling. 

## Conflict of interest

The authors declare that the research was conducted in the absence of any commercial or financial relationships that could be construed as a potential conflict of interest.

## Publisher’s note

All claims expressed in this article are solely those of the authors and do not necessarily represent those of their affiliated organizations, or those of the publisher, the editors and the reviewers. Any product that may be evaluated in this article, or claim that may be made by its manufacturer, is not guaranteed or endorsed by the publisher.
